# EARLY COGNITIVE DECLINE IN AMYOTROPHIC LATERAL SCLEROSIS AND ITS RELATION TO DRIVING: AN OBSERVATIONAL STUDY

**DOI:** 10.2340/jrm.v57.43483

**Published:** 2025-09-24

**Authors:** Tina TAULE, Ole-Bjørn TYSNES, Jörg AßMUS, Annbjørg SPILDE MORLAND, Marit ARNEVIK RENSÅ, Tone REVHEIM, Synnøve GLESNES, Tiina REKAND

**Affiliations:** 1 Department of Occupational Therapy, Orthopedic Clinic, Haukeland University Hospital, Bergen, Norway; 2 Western Norway University of Applied Sciences, Bergen, Norway; 3 Department of Neurology, Neurologic Clinic, Haukeland University Hospital, Bergen, Norway; 4 Centre for Clinical Research, Haukeland University Hospital, Bergen, Norway; 5 Institute of Neuroscience and Physiology, Sahlgrenska Academy, University of Gothenburg, Gothenburg, Sweden

**Keywords:** activities of daily living, amyotrophic lateral sclerosis, cognition, driving, neurology, neuropsychological test

## Abstract

**Objective:**

To determine whether early cognitive function in amyotrophic lateral sclerosis patients predicts future cognitive function and the decision to cease driving.

**Design:**

Observational study.

**Subjects:**

Patients with amyotrophic lateral sclerosis.

**Methods:**

Subjects underwent baseline assessments of cognitive function and driving ability within 4 months of diagnosis, with follow-up evaluation conducted 4 months thereafter. Two hypotheses were tested: (H1) cognitive status remains stable between baseline and follow-up, (H2) patients with baseline cognitive impairment cease driving earlier than those without cognitive changes. Data were analysed using *t*-tests and regression analysis, with visual inspection of the results.

**Results:**

Of 31 subjects tested at baseline, 5 were under 60 years old, 11 were female, 11 were cognitively impaired, and 61% were driving. Over the 4-month period, cognitive function of the subjects (*n* = 21) did not change significantly. There was no significant association between baseline cognitive function and follow-up driving status.

**Conclusion:**

Early cognitive function assessment in amyotrophic lateral sclerosis predicts future cognitive function but not currently the decision to cease driving. Cognitive impairment occurs early in the disease, highlighting the importance of early evaluation and implementation of safety measures related to driving.

## LAY ABSTRACT

This study investigated whether early cognitive function in patients with amyotrophic lateral sclerosis could predict future cognitive functioning and its impact on the decision to cease driving earlier than those without cognitive changes. We followed a group of amyotrophic lateral sclerosis patients over time. Participants did cognitive tests and driving ability assessment up to 4 months from diagnosis, and follow-up evaluation up to 8 months after being diagnosed. Although those with early cognitive impairment consistently showed lower cognitive scores, this did not predict their decision to stop driving. In conclusion, early cognitive testing in patients with amyotrophic lateral sclerosis can help predict future cognitive function but does not influence current driving decisions. Cognitive impairment appears early in the disease, emphasizing the need for early evaluation. Identifying cognitive changes early enables healthcare professionals to provide better advice and focus on driving safety, helping patients maintain their independence and stay safe.

Amyotrophic lateral sclerosis (ALS) is a fatal neurodegenerative multisystem disease with a typical survival of 2 to 5 years (1). In Europe, ALS has an incidence of 2.3 per 100,000 persons per year, and a prevalence of 6.2 per 100,000 individuals (2). While ALS primarily affects motor function (1), cognitive impairment due to frontotemporal dysfunction occurs in 30% to 75% of patients (3). Research indicates that cognitive deficits may emerge early in the course of ALS, potentially even before motor symptoms manifest (4). However, further investigation is needed on the progression of cognitive deficits (4).

ALS-specific cognitive impairments are heterogenous, most commonly affecting attention, executive function, language, and verbal memory (3, 4). These cognitive functions are crucial for planning and complex problem-solving (5, 6), which may impact several activities, including navigating traffic and driving. However, the relationship between cognitive impairment and driving in ALS patients is poorly studied (7), and there is limited attention to how these cognitive challenges are addressed in meetings between healthcare professionals, patients, and carers.

While motor function is often assessed in relation to driving ability in ALS patients, cognitive changes are frequently overlooked (8). This neglect may lead to incomplete evaluations, posing a risk to both the driver and others in traffic. Although not all individuals with cognitive impairment are unsafe drivers, it is crucial to distinguish those who can drive safely from those who should cease driving (9). Concerningly, common tools used to assess cognitive function in ALS patients may be unsuitable for this purpose (8). Cognitive impairment in ALS differs from other types of dementia, which are typically assessed using clinically approved scales like the Mini-Mental State (MMSE) or the Montreal Cognitive Assessment (MoCA). Accurate identification of cognitive impairment in ALS requires purposeful evaluation using ALS-specific measures that account for the motor difficulties commonly associated with the disease (10). As a first step to identify those with and without cognitive challenges, as well as determining the types and severity of cognitive difficulties present, initial screening with use of ALS-specific screening instruments is widely recommended (10).

Without a clear understanding of how cognitive difficulties influence driving ability in ALS patients, managing these challenges becomes difficult and uncertain (11). Additionally, driving is a crucial daily activity that enhances independence and well-being (12). This creates a dilemma for healthcare professionals, who must balance ensuring safety with the significant benefits that driving provides to ALS patients. The need to address this issue early in the disease adds complexity, as multiple urgent topics demand attention.

The aims of this study are to evaluate whether cognitive function early in the course of ALS predicts future cognitive problems and earlier cessation of driving. By addressing these aims, the study seeks to provide valuable insights into how cognitive function affects ALS patients over time and impacts their ability to perform daily activities such as driving, informing clinical practice and guiding future research.

We formulated 2 hypotheses: (H1) cognitive status in patients with ALS will not change significantly over the 8 months following diagnosis; (H2) patients with cognitive impairment at baseline will cease driving earlier than those without cognitive impairments (13).

## METHODS

### Study design

This observational study investigated the characteristics and treatments of ALS patients in a real-world setting, as guided by Camm and Fox (14). To ensure transparency, we report our study according to recommendations for observational studies (STROBE) (Table SI).

### Protocol and registration

This study was registered at ClinicalTrials.gov (registration number NCT03578796; available at https://clinicaltrials.gov/study/NCT03578796). The protocol has been published by Taule et al. (13).

### Participants

Subjects were recruited from the ALS outpatient clinic at Haukeland University Hospital within months of their ALS diagnosis, between 30 April 2017, and 1 May 2021. Recruitment was conducted by a member of our ALS-specific healthcare team in conjunction with the patients’ first clinic visit. To ensure equal understanding of tasks, participants who were native Norwegian speakers were included. We excluded ALS patients who had severe writing or reading difficulties, as well as those with comorbid conditions affecting cognitive function. The exclusions were necessary to avoid confusion regarding results derived from the cognitive test. Each included ALS subject chose 1 carer to assist him or her in the study.

### Data collection

Participants underwent a standardized programme of tests that, according to the International Classification of Functioning, Disability and Health (ICF) (15), assessed mental functions and driving ability. Mental function was screened using the Edinburgh Cognitive and Behavioural ALS Screen (ECAS) (16), with assessment carried out by occupational therapists and a nurse. Participants’ driving ability was assessed using a modified questionnaire adapted from the Norwegian ParkWest study (17). Clinicians, who were blinded to cognitive test scores, conducted the driving assessments. These tests were performed up to 4 months from diagnosis (baseline) and the retest (follow-up) was done up to 8 months after being diagnosed. Occasionally, follow-up was not possible because participants declined, were placed on permanent ventilation support, or had passed away. Baseline demographic characteristics including age, gender, and highest level of education attained were collected using a custom-designed questionnaire. Baseline physical and medical function were assessed with the ALS Functioning Rating Scale–revised version (ALS-FRS-R) (18).

### Edinburgh Cognitive and Behavioural ALS Screen

Baseline ECAS scores were used as a predictor variable for incident cognitive impairment and cessation of driving. The ECAS is a brief, well-established screening test for detecting cognitive and behavioural changes in patients with ALS (16). It was designed to accommodate physical disabilities that often accompany ALS; that is, spoken or written responses can be accepted (16).

The component of the ECAS called the ECAS–cognitive screen comprises an ALS-specific sub-score (0–100) for language, verbal fluency, and executive functions, and an ALS-nonspecific sub-score (0–36) for memory and visuospatial functions, leading to a total score (0–136) (16). Higher scores indicate better cognitive function (16). The ECAS was translated into Norwegian (ECAS-N) in collaboration with the University of Edinburgh, Scotland (registration number SC005336). The cut-off value for the cognitive screen of ECAS-N total score (< 92 points) indicated cognitive impairment (19).

The component of the ECAS called the ECAS–behavioural screen comprises 5 behavioural domains and 3 questions related to psychosis (16). It is a carer interview designed to assess changes in a patient’s performance in these areas from the perspective of the carer. Score ranges from 0–10 for behaviour changes, and 0–3 for psychosis, with higher scores indicating greater impairment (16). The ECAS-N is found valid when used by different testers, and for documenting cognitive function over time (20).

### Driving ability

Participants’ driving ability was assessed using yes/no answers and elapsed time to demonstrate reduced function, from the Norwegian ParkWest study (17). The questions posted were: (*i*) Do you have a driving licence? and (*ii*) Do you currently drive a car?

### ALS-FRS-R

The ALS-FRS-R evaluate patients’ different activities, such as ability to speak, swallow, perform daily activities, and breathe using a 12-item questionnaire scored from 0 (worse) to 4 (best), with a total score of 48 indicating high function (18).

### Sample size

From 2012 to 2017, our clinic diagnosed 12 to 15 ALS patients annually. Based on this census, we expected to recruit about 50 to 60 patients over the 4-year study period.

### Statistical analyses

Data were analysed using SPSS version 26.0 (SPSS 2019) (IBM Corp, Armonk, NY, USA); R 4.2.0 (R Foundation for Statistical Computing, Vienna, Austria) (21); and MATLAB version 9.0 (https://www.mathworks.com/products/matlab.html) (22). Due to minimal variation in the data, the ECAS-N psychosis score was excluded from the analysis. Descriptive statistics were used to characterize the sample at baseline. Changes in ECAS-N scores between assessments were evaluated by paired *t*-tests, and their association was assessed using linear regression models and graphical tools (H1). As data for driving variable were much skewed, we decided not to perform inference tests, and instead used only graphical tools to assess H2. The association between baseline ECAS-N scores and time to cease driving was analysed using a Cox proportional hazard model. We did not have sufficient data to adjust the regression model; this mainly affected the evaluation of H2. *P*-values < 0.05 were considered significant.

### Ethics

The Regional Ethical Research Committee-West approved this study (reference number 2016/2187/REK west). Informed consent was obtained from all participants, and the study adhered to the World Medical Association Declaration of Helsinki (23).

## RESULTS

**Fig. 1:**
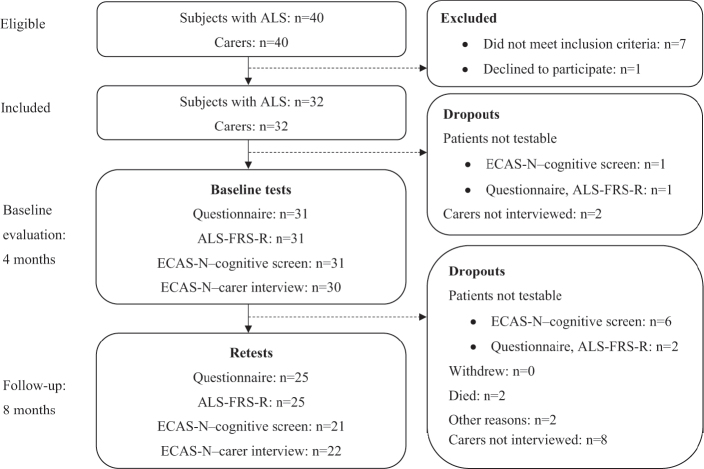
Flow diagram of subjects with ALS. ALS: amyotrophic lateral sclerosis; ALS-FRS-R: Amyotrophic Lateral Sclerosis-Functional Rating Scale–revised; ECAS-N: Edinburgh Cognitive and Behavioural ALS Screen–Norwegian version.

Fig. 1 presents a flow diagram showing subject selection procedures, tests administered, and the timeline for tests and follow-up assessments.

The baseline demographic characteristics of the 31 subjects who underwent baseline evaluation are given in Table I. The median time from diagnosis to the baseline test was 65 days (range: 1–119 days). The median interval between diagnosis and the retest was 213 days (range: 146–246 days). Most of the subjects were 60 years or older at baseline, and most were men. Approximately 60% were driving a car regularly at the time of baseline assessment.

**Table I:** Baseline demographic characteristics of subjects with amyotrophic lateral sclerosis (ALS)

Variables	Subjects with ALS (*n* = 31)
Gender, female, *n* (%)	11 (36%)
Age, < 60 years, *n* (%)	5 (19%)
Civil status, living with a partner, *n* (%)	23 (74%)
Highest educational level attained, *n* (%)	
Elementary school up to high school	15 (48%)
Higher education and/or university education	16 (52%)
Driving status	
Possess driver’s licence for car	25 (81%)
Drive a car regularly	19 (61%)

The baseline clinical characteristics of the 31 subjects who underwent baseline evaluation are presented in Table II. Eleven subjects (36%) were cognitively impaired, as indicated by their baseline ECAS-N scores. Among these, 7 subjects were driving a car regularly. At the follow-up, 25 subjects (81%) remained in the study. All of these subjects were reassessed with the ALS-FRS-R and the modified questionnaire; 21 were testable with the ECAS–cognitive screen, and 22 with the ECAS–behavioural screen.

**Table II:** Baseline clinical characteristics of subjects with amyotrophic lateral sclerosis (ALS)

Variables	Subjects with ALS (*n* = 31)
ALS-FRS-R score, mean (SD)	39 (7)
ECAS-N–cognitive score, mean (SD)	
Total score	101 (13)
ALS-specific score	74 (11)
ALS-nonspecific score	27 (4)
ECAS-N–behavioural score, median (min, max)	1 (0, 8)^a^

a One missing ECAS-N–behavioural score.

ALS-FRS-R: amyotrophic lateral sclerosis-functional rating scale-revised; ECAS-N: Edinburgh Cognitive and Behavioural ALS Screen – translated Norwegian version; SD: standard deviation.

### Results for H1

**Fig. 2:**
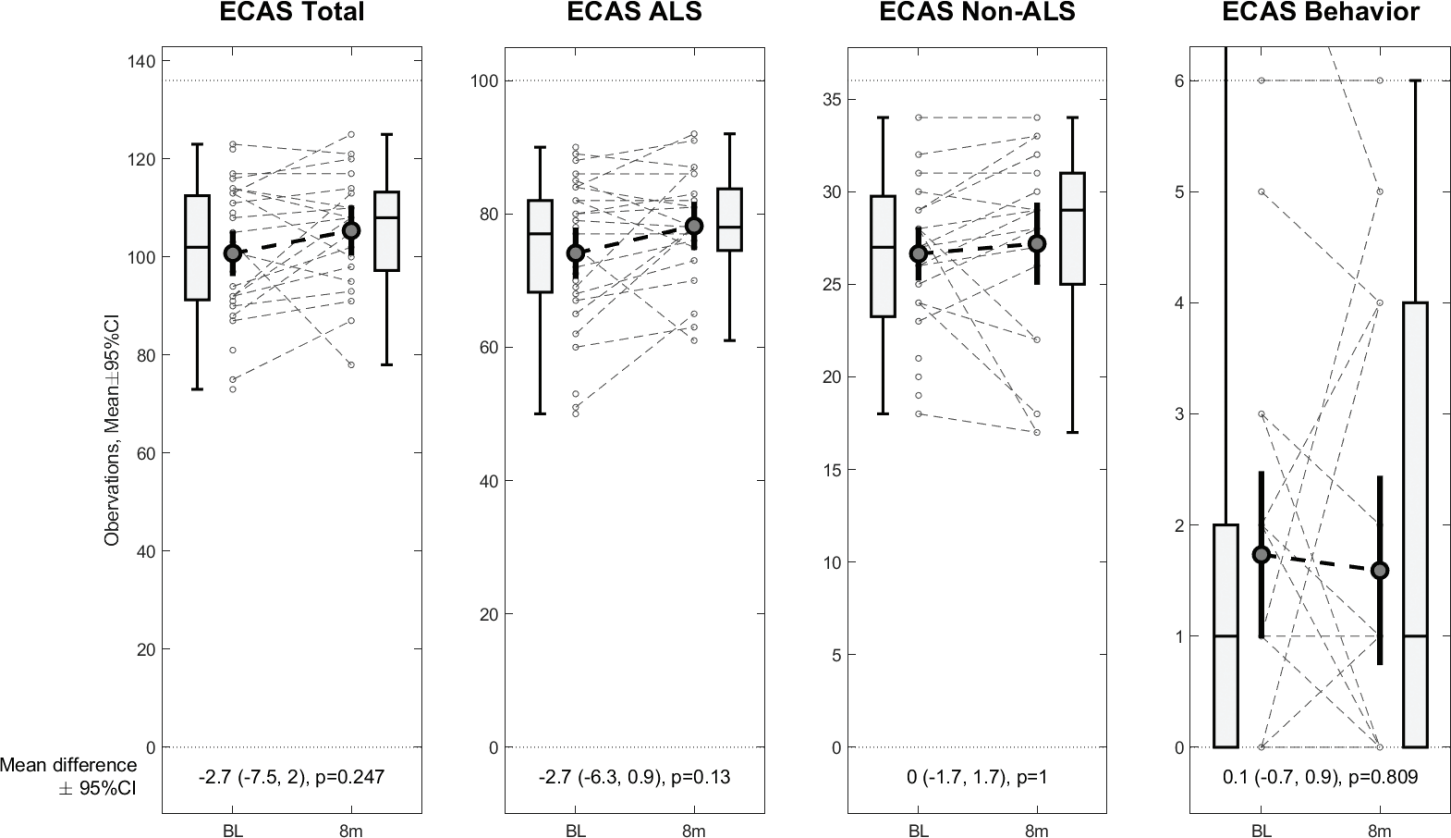
Box plot of the distribution of ECAS-N scores for ALS subjects at baseline and follow-up.

We hypothesized that the cognitive status of ALS patients would not change significantly between baseline assessment and the follow-up, as measured by the ECAS-N. For H1, we found that subjects’ cognitive function did not differ significantly from baseline to follow-up (Fig. 2). There was a significant association between cognitive function at baseline and cognitive function at the follow-up. These results indicate that subjects’ cognitive status remained stable during the time period between baseline and follow-up assessment.

### Results for H2

We hypothesized that ALS patients who were cognitively impaired at baseline, as assessed by the ECAS-N, would cease driving earlier than those without cognitive impairment. For H2, we found no association between patients’ baseline cognitive function and their driving status (yes/no) at last observation. Whether a subject was cognitively impaired at baseline did not affect their decision to continue driving (Table III). Thus, the results did not support H2. At the follow-up, 22 subjects still possessed a driving licence, and of these, 13 continued to drive regularly. Two of the subjects who continued to drive at follow-up exhibited signs of cognitive impairment.

**Table III:** Cox regression analysis of associations between baseline scores on the ECAS-N and car driving at last observation

Outcomes	*n*	HR (95% CI)	*p*-value
ECAS-N			
ALS-specific score	29	0.96 (0.87, 1.05)	0.344
ALS-nonspecific score	29	0.82 (0.58, 1.16)	0.268
Total score	29	0.96 (0.88, 1.04)	0.287
Behaviour score	28	0.59 (0.21, 1.68)	0.322

ALS: amyotrophic lateral sclerosis; CI: confidence interval; ECAS-N: Edinburgh Cognitive and Behavioural ALS Screen – translated Norwegian version; HR: hazard ratio.

**Fig. 3:**
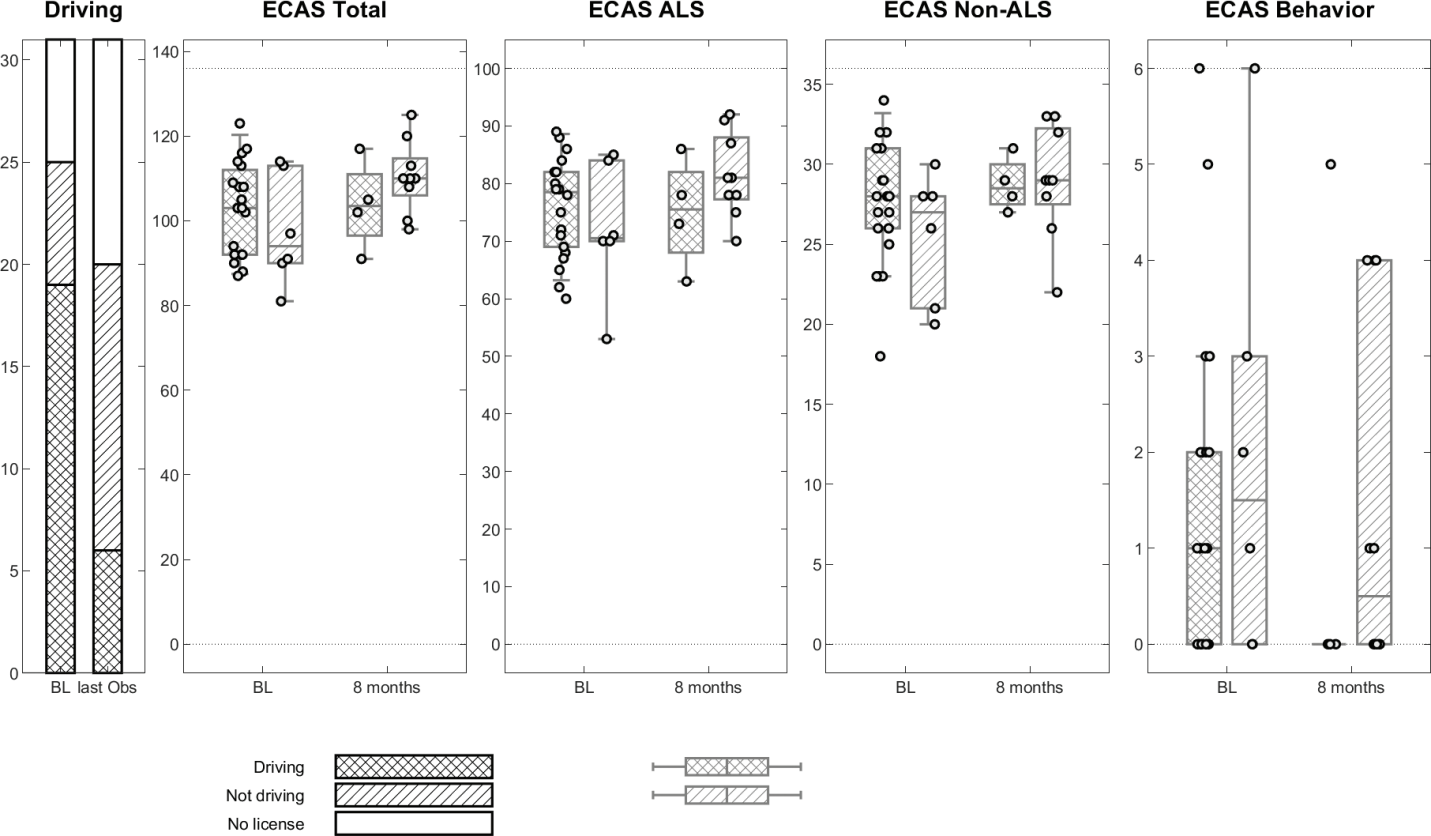
Box plot of the distribution of ECAS-N scores for driving and not-driving ALS subjects at baseline and follow-up. Abbreviations: ALS: amyotrophic lateral sclerosis; ECAS-N: Edinburgh Cognitive and Behavioural ALS Screen–Norwegian version.

The visualization of results depicted in Fig. 3 indicates a slight difference in cognitive function between subjects who continued driving and those who stopped driving.

## DISCUSSION

This study shows that cognitive impairment remains stable during the time period between baseline and follow-up assessments and did not influence the patients’ decisions to cease driving.

### Cognitive impairment did not influence decisions to cease driving

Our finding that cognitive impairment in ALS appears not to be associated with the decision to cease driving rejected H2 and indicates several possibilities. Either driving ability changes little over the assessed period, or declines in driving fitness are not being recognized or fully appreciated by patients or clinicians. Alternatively, it is possible that appropriate advice regarding driving is provided during consultations but not being followed by the subjects.

Supporting the notion of stability in driving fitness, a study by Hayes et al. (24) tested subjects with ALS and healthy controls in a driving simulator and found no significant differences in performing driving tasks between the 2 groups. However, the cognitive status of the ALS patients in that study was not assessed.

Our result may reflect the reality of clinical practice, where brief consultations with numerous pressing topics on the agenda often leave driving capability underexplored. From the patient’s perspective, driving offers freedom and independence, essential for well-being, social life, and mental health (12). It remains unclear how patients perceive their driving abilities as their cognitive function declines and what they communicate concerning these perceptions during their consultations (6). Healthcare professionals often struggle to identify the impact of cognitive changes on patients’ daily life (5, 6, 24), such as driving. This difficulty may stem from distinguishing cognitive changes from psychological responses to a terminal diagnosis and progressive physical impairment (6). Additionally, motor disability may mask cognitive difficulties (25). Therefore, addressing the cognitive challenges of ALS patients and their implications for daily life must be prioritized during consultations (26). Ensuring personal independence and safety for those involved in activities like driving is crucial.

Failure to detect or fully appreciate impaired cognitive function may pose a serious threat to driving safety. This topic, while critically important, is scarcely reported in studies of ALS patients. A literature review on motor vehicle safety in patients with frontotemporal dementia found that these patients had more hit-and-run accidents, frequently failed to stop at red lights, incurred more speeding infractions, and failed to recognize pedestrians at intersections, compared with healthy controls (27). The authors attributed these impairments to poor impulse control, impaired planning ability, poor problem-solving skills, poor judgement, and difficulty making appropriate decisions (27). Similar cognitive problems are also commonly reported in patients with ALS (6, 28, 29).

Routine screening of patients’ cognitive function is a recommended first step (10) in identifying ALS patients who should be advised to stop driving and those in need of more tailored intervention programmes. A relatively large proportion of participants (36%) in our study had baseline ECAS scores indicative of cognitive decline, suggesting that cognitive impairment may be present early in the course of ALS. That similar finding is reported in a meta-analysis by Finsel et al. (4) underscores the importance of early screening by use of ALS-specific tools like the ECAS. Finsel et al. (4) also showed that cognitive impairment may occur even before motor impairment, and highlight the importance of equating the patient’s cognitive status with motor difficulties in what should be a mandatory conversation about driving. To prevent accidents, healthcare professionals must increase their focus on driving ability among ALS patients. Physicians should take special responsibility to include driving ability as a routine topic during consultations. Conversations with patients may include modifications to help continue driving, driving safety from the perspective of patients and family members, alternative transport options, and, ideally, an in-person driving fitness test. A previous study showed that ALS subjects with cognitive problems rated their quality of life highly, possibly indicating a lack of insight into their own situation (30). Healthcare professionals, particularly physicians, are responsible for adjusting patient expectations in accordance with their cognitive and motor function declines.

### Cognitive impairment remains stable over time

Our finding of stable cognitive function over time confirmed H1 and aligns with those reported in a meta-analysis of cognitive progression in ALS, which included data from both motor-independent measures and those not specifically designed for ALS (4). Similar to our study, 3 longitudinal studies included in the meta-analysis by Finsel et al. (4) used the ALS-specific measure, ECAS, to assess cognition over time (25, 31, 32). These studies reported no significant deterioration in ECAS scores for up to 18 months post-diagnosis. One explanation for the stable ECAS scores during the early stages of the disease may be that cognitive deterioration is masked by a learning effect from repeated measurements. However, Burkhardt et al. (25), investigated this phenomenon and found no practice effect in ALS subjects for up to 18 months post-diagnosis. Although Poletti et al. (31) reported some improvement in ECAS scores and suggested learning effect as a possible explanation, this occurred late in the disease course and after 3 evaluations. A more plausible explanation of the stable ECAS scores is that pre-symptomatic cognitive deterioration may be captured by baseline testing, but does not decline further during the early months of the disease. This is also suggested by Finsel et al. (4). Taken together, our results and previous research imply that a patient’s cognitive prognosis may be predicted using the baseline score. However, the evidence for the progression of cognitive deficits is still limited, and the absence of significant change in cognitive performance during our short assessment period does not preclude future changes as the disease progresses.

### Strengths and limitations of the study

The current study had several strengths. The ECAS is a well-established instrument with known psychometric characteristics (33). The study also benefited from involving a biostatistician during the statistical analysis and ensuring all test administrators were ECAS certified. At the 8-month follow-up, a relatively high percentage (81%) of subjects remained in the study, indicating a robust follow-up rate.

Our study indicates a slightly higher proportion of men (64%), which aligns with the reported male-to-female ratio of 1 and 2 to 1 (34). The majority of our participants were over 60 years old (*n* = 26), reflecting the later age at ALS onset that has been reported in European countries (34). Additionally, the finding of cognitive decline in 36% of the patients closely matches cautious estimates of the prevalence of cognitive decline in ALS (3). These factors suggest that our sample is representative of the general ALS patient population, as it reflects known demographic and clinical characteristics typical of this group. However, the study also had some limitations. First, the sample size was small, which typically results in low statistical power. Given that ALS is a rare disease, recruiting large samples is challenging. A multicentre study can change this.

To mitigate potential practice effects in repeatedly administering the ECAS, it is recommended to use alternative forms for repeated measures designs (35). However, an alternative form of the ECAS-N is not yet available. Therefore, the time interval between baseline and retest administration was carefully chosen. It was considered long enough to minimize recall bias but short enough to reduce the possibility of study withdrawal due to symptom worsening. Previous research also indicated that the constructs measured by the ECAS are stable over our study period (25).

Future studies should incorporate additional information, such as records of crashes and infractions, practical evaluation of driving skills, and inspections of the patient’s car for dents and scratches that might indicate difficulties with driving and parking. This information, along with interviews of family members, would help determine whether patients with ALS should continue driving. Additionally, because some patients live with ALS for some time before being diagnosed, future studies should include information concerning the stage of the disease at the time of diagnosis.

Despite the aforementioned limitations, the present work presents the first follow-up study of Norwegian patients assessed with the ECAS-N, which many consider to be the new gold standard for cognitive screening in Norwegian ALS-specific outpatient clinics.

### Conclusion

The cognitive status of ALS subjects in our study was stable during the time period between baseline and follow-up assessment and the patients’ cognitive function at follow-up was predicted by their cognitive function early in the course of ALS. These results indicate that the patient’s cognitive prognosis may be predicted at the first scoring by use of the ECAS-N, which means that 1 screening, conducted early in the course of ALS, provides healthcare professionals with the information they need to act. Cognitive function was not associated with driving status in this study. This means that healthcare professionals must address this issue in consultations with ALS patients and cognitive status must be evaluated. Cognitive impairment may be present in a very early stage of the disease, and may be present before the decline in motor skills. The ECAS-N is a suitable screening test to reveal those in need of tailored communication and interventions. There is a need for longer term follow-up studies to observe cognitive function changes over a more extended period of time.

## Supplementary Material


