# Supplementary material for: EARLY COGNITIVE DECLINE IN AMYOTROPHIC LATERAL SCLEROSIS AND ITS RELATION TO DRIVING: AN OBSERVATIONAL STUDY
Source: J Rehabil Med. 2025 Sep 24;57:43483. doi: 10.2340/jrm.v57.43483 (PMC12490105; doi:10.2340/jrm.v57.43483)
Supplement: Supplementary file 1 [file JRM-57-43483-s1.pdf]

Supplementary material has been published as submitted. It has not been copyedited, or typeset by Journal of Rehabilitation Medicine

Table SI. STROBE Statement—checklist of items that should be included in reports of observational studies

	Item No.	Recommendation	Page No.	Relevant text from manuscript
**Title and abstract**	1	(*a*) Indicate the study's design with a commonly used term in the title or the abstract	1	Early cognitive decline in Amyotrophic Lateral Sclerosis and its relation to driving. An observational study
		(*b*) Provide in the abstract an informative and balanced summary of what was done and what was found	3	*Objective:* To determine whether early cognitive function in amyotrophic lateral sclerosis patients predicts future cognitive function and the decision to cease driving.<br>*Design:* Observational study<br>*Subjects:* Patients with amyotrophic lateral sclerosis<br>*Methods:* Subjects underwent baseline assessments of cognitive function and driving ability within four months of diagnosis, with follow-up evaluation conducted four months thereafter. We tested two hypotheses: (H1) cognitive status remains stable between baseline and follow-up, (H2) patients with baseline cognitive impairment cease driving earlier than those without cognitive changes. Data were analysed using *t*-tests and regression analysis, with visual inspection of the results.<br>*Results:* Of 31 subjects tested at baseline, 5 were under 60 years old, 11 were female, 11 were cognitively impaired, and 61% were driving. Over the four-month period, cognitive function of the subjects' (n=21) did not significantly change. There was no significant association between baseline cognitive function and follow-up driving status.

				Conclusion: Early cognitive function assessment in amyotrophic lateral sclerosis predicts future cognitive function but not currently the decision to cease driving. Cognitive impairment occurs early in the disease, highlighting the importance of early evaluation and implementing safety measures related to driving.<br><br>**Clinical Trials Registration:** NCT03578796.
**Introduction**				
Background/rationale	2	Explain the scientific background and rationale for the investigation being reported	5-6	There are a significant knowledge gap on the progression of cognitive deficits in ALS. Additionally, the relationship between cognitive impairment and driving in ALS patients is poorly studied, and there is limited attention to how these cognitive challenges are addressed in meetings between healthcare professionals, patients and carers.<br><br>The scientific background and rationale for the study is further elaborated in the Introduction section of the manuscript.
Objectives	3	State specific objectives, including any prespecified hypotheses	6	The **objectives** of this study were to evaluate whether cognitive function early in the course of ALS predict future cognitive problems and earlier cessation of driving.<br><br>We formulated two **hypotheses**: (H1) cognitive status in patients with ALS will not change significantly over the eight months following diagnosis; (H2) patients with cognitive impairment at baseline will cease driving earlier than those without cognitive impairments.
**Methods**				

Study design	4	Present key elements of study design early in the paper	6	Title: Early cognitive decline in Amyotrophic Lateral Sclerosis and its relation to driving. An observational cohort study
Setting	5	Describe the setting, locations, and relevant dates, including periods of recruitment, exposure, follow-up, and data collection	6-7	**Setting:** Real-world setting<br>**Location:** ALS outpatient clinic at an university hospital<br>**Periods of recruitment:** Between April 30, 2017, and May 1, 2021<br>**Exposure:** NA<br>**Follow-up:** within 4 months after being diagnosed (baseline) and follow-up within 8-months after being diagnosed<br>**Data collection**: Participants underwent a standardised programme of tests at baseline and follow-up.
Participants	6	(*a*) ***Cohort study***—Give the eligibility criteria, and the sources and methods of selection of participants. Describe methods of follow-up<br>*Case-control study*—Give the eligibility criteria, and the sources and methods of case ascertainment and control selection. Give the rationale for the choice of cases and controls<br>*Cross-sectional study*—Give the eligibility criteria, and the sources and	7	**Eligible criteria:** To be eligible as subjects, patients had to have sought care at the ALS outpatient clinic at our hospital within four months after being diagnosed with ALS. Between April 30, 2017, and May 1, 2021.<br>**Sources of selection of participants:** A member of our ALS-specific healthcare team recruited study participants in conjunction with their first visit to the hospital.<br>**Methods of selection of participants:** Only participants who were native Norwegian speakers were included. We excluded ALS patients who had great difficulties in writing or reading, as well as those with comorbidities in which cognitive function was impaired. The exclusions were necessary to avoid confusion about results derived from the cognitive test. Each included ALS subject also chose one carer to assist him or her in the study.

		methods of selection of participants		**Methods of follow-up:** Participants underwent a standardised programme of tests within 4 months after being diagnosed with ALS (baseline) and follow-up within 8-months after being diagnosed. Cognitive tests were done along with clinical evaluations.
		(*b*) *Cohort study*—For matched studies, give matching criteria and number of exposed and unexposed<br>*Case-control study*—For matched studies, give matching criteria and the number of controls per case		NA
Variables	7	Clearly define all outcomes, exposures, predictors, potential confounders, and effect modifiers. Give diagnostic criteria, if applicable	7-8	**Outcomes:** Cognitive function, physical and medical information, ability to drive a car<br>**Predictor:** Cognitive function at baseline<br>**Confounders:** Age, gender, civil status, highest level of education attained<br>**Effect modifiers:** NA
Data sources/ measurement	8*	For each variable of interest, give sources of data and details of methods of assessment (measurement). Describe	*8-10*	**Sources of data and details of methods of assessment**

		comparability of assessment methods if there is more than one group		• **Cognitive function:** At baseline and follow-up, a cognitive test were done along with clinical evaluations and assessed by use of **the translated Norwegian version of Edinburgh Cognitive and Behavioural ALS Screen (ECAS-N).**<br>• **Physical and medical information** at baseline and follow-up were assessed with the **ALS Functioning Rating Scale–revised version**<br>• **Participants' ability to drive a vehicle** were assessed with a **modified questionnaire** that was originally used in the Norwegian ParkWest study<br>• **Background information** was obtained with a **custom-designed questionnaire**<br>All outcomes and the predictor are further outlined in the Method section.<br><br>**Comparability of assessment methods:** All participants underwent the same standarised programme of tests.
Bias	9	Describe any efforts to address potential sources of bias	17	Efforts to address potential sources of bias<br>• Using well established instruments with known psychometric characteristics<br>• Using a biostatistician during the statistical analysis<br>• Using certified test administrators<br>• Carefully selecting a time interval between baseline and retest considered long enough to minimize recall bias but short enough to reduce the possibility of study withdrawal due to symptom worsening
Study size	10	Explain how the study size was arrived at	9	Annually from 2012 to 2017, 12 to 15 patients were diagnosed with ALS at our clinic. Based on this census, a total of about 50 to 60 patients were expected to be included in the current study over a four-year period.

Quantitative variables	11	Explain how quantitative variables were handled in the analyses. If applicable, describe which groupings were chosen and why	10	Data were analysed using SPSS version 26.0 (18); R 4.2.0 (19); and MATLAB version 9.0 (20). Due to minimal variation in the data, the ECAS-N psychosis score was not included in the present study's analysis.
Statistical methods	12	(*a*) Describe all statistical methods, including those used to control for confounding	10	• Descriptive statistics<br>• t-tests<br>• Linear regression models<br>• Graphical evaluation<br>• Cox proportional hazard model
		(*b*) Describe any methods used to examine subgroups and interactions	10	Descriptive statistics were used to characterise the sample at baseline. Changes in ECAS-N scores between assessments were evaluated by paired *t*-tests, and their association was assessed using linear regression models as well as graphically (H1). Since data for driving variables were much skewed, we decided not to perform inference tests, and instead used only graphical tools to assess H2. The association between ECAS-N at baseline and elapsed time to cease driving was assessed using a Cox proportional hazard model. P-values less than 0.05 were considered significant.
		(*c*) Explain how missing data were addressed	Page 10 Table 1-3<br>Fig 1	If missing, this is reported in Table 1-3. The reasons for non-response are reported in Figure 1. Missing data are not included in the analysis.
		(*d*) *Cohort study*—If applicable, explain how loss to follow-up was addressed		This study was not extended with follow-up study.

		Case-control study—If applicable, explain how matching of cases and controls was addressed<br><br>*Cross-sectional study*—If applicable, describe analytical methods taking account of sampling strategy		
		(*e*) Describe any sensitivity analyses		Sensitivity analyses are not performed
**Results**				
Participants	13*	(a) Report numbers of individuals at each stage of study—eg numbers potentially eligible, examined for eligibility, confirmed eligible, included in the study, completing follow-up, and analysed	Figure 1, Table 1-3<br><br>Figure 2-3	Number of subjects with ALS and Carers<br>• Eligible: subjects with ALS (n=40), Carers (n=40)<br>• Included: subjects with ALS (n=32), Carers (n=32).<br>• Baseline evaluation: ECAS-N cognitive screen (n=31), ECAS-N behavioural screen (n=30), ALS-FRS-R (n=31), car driving (n=31).<br>• Evaluation: ECAS-N cognitive screen (n=21), ECAS-N behavioural screen (n=22), ALS-FRS-R (n=25), car driving (n=25)<br>• Analysed: Descriptive statistics at baseline: n=31/30; t-test: n=21/22; Linear regression models: n= 21/22; Cox regression analysis: n=29/28
		(b) Give reasons for non-participation at each stage		• Eligible to inclusion: Seven patients did not meet inclusion criteria and 1 declined to participate.<br>• Inclusion to baseline test: One patient dropped out of the study before baseline test<br>• Baseline test to follow-up: Six patients were not testable at follow-up, 2 died between baseline and follow-up and 2 patients drop out of the study for other reasons.

		(c) Consider use of a flow diagram	Fig 1	A flow-diagram is provided.
Descriptive data	14*	(a) Give characteristics of study participants (eg demographic, clinical, social) and information on exposures and potential confounders	Table 1	Demographic information is provided in Table 1. Clinical information is provided in Table 2.
		(b) Indicate number of participants with missing data for each variable of interest	Table 1-3	Participants with missing data is provided for each variable of interest
		(c) *Cohort study*—Summarise follow-up time (eg, average and total amount)	Table 1-3	The participants were followed maximally until 8 months after being diagnose or until death.
Outcome data	15*	*Cohort study*—Report numbers of outcome events or summary measures over time	Table 1-3	The number of observations and SD are reported. The association between ECAS-N at baseline and elapsed time to cease driving was assessed using a Cox proportional hazard model
		Case-control study—Report numbers in each exposure category, or summary measures of exposure		
		Cross-sectional study—Report numbers of outcome events or summary measures		
Main results	16	(*a*) Give unadjusted estimates and, if applicable, confounder-adjusted estimates and their precision (eg, 95% confidence interval). Make clear which		

		confounders were adjusted for and why they were included		
		(*b*) Report category boundaries when continuous variables were categorized	8	The participants were divided into two groups, cognitive impaired (cut-off value for ECAS-N total score (≤ 92 points) and not cognitive impaired (ECAS-N total score > 92 points).
		(*c*) If relevant, consider translating estimates of relative risk into absolute risk for a meaningful time period		
Other analyses	17	Report other analyses done—eg analyses of subgroups and interactions, and sensitivity analyses		
**Discussion**				
Key results	18	Summarise key results with reference to study objectives	13	Early cognitive function assessment in amyotrophic lateral sclerosis predicts future cognitive function but not currently the decision to cease driving. Cognitive impairment occurs early in the disease, highlighting the importance of early evaluation and implementing safety measures related to driving.
Limitations	19	Discuss limitations of the study, taking into account sources of potential bias or imprecision. Discuss both direction and magnitude of any potential bias	17	Two limitations are that the study had some loss to follow-up, and the sample size was small; smaller sample size typically mean low statistical power. Additionally, a potential practice effect in repeatedly administering the ECAS-N without using an alternate form of the ECAS-N is a possible bias of the study. A possible bias is also related to the study participants found to differ slightly from cohorts in comparable studies.

Interpretation	20	Give a cautious overall interpretation of results considering objectives, limitations, multiplicity of analyses, results from similar studies, and other relevant evidence	16 and 18	In conclusion, the cognitive status of ALS subjects in our study was stable during the time period between baseline and follow-up assessment and the patients' cognitive function at follow-up were predicted by their cognitive function early in the course of ALS. These results indicate that the patient's cognitive prognosis may be predicted at the first scoring by use of the ECAS-N, which means that one screening, conducted early in the course of ALS provides healthcare professionals the information they need to act. Cognitive function was not associated with driving status in this study. This means that healthcare professionals must address this issue in consultations with ALS patients and cognitive status must be evaluated. Cognitive impairment may be present in very early stage of disease, and may before the decline of motor skills. The ECAS-N is a suitable screening test to reveal those in need of tailored communication and interventions. There is a need for longer-term follow-up studies to observe cognitive function changes over a more extended period of time.
Generalisability	21	Discuss the generalisability (external validity) of the study results	18	Loss to follow up, and the small sample size typically mean low statistical power.
**Other information**				
Funding	22	Give the source of funding and the role of the funders for the present study and, if applicable, for the original study on which the present article is based	18	This work was supported by the Western Norwegian Regional Health Authority under Grant number 912158, 2017.

*Give information separately for cases and controls in case-control studies and, if applicable, for exposed and unexposed groups in cohort and cross-sectional studies.

Note: An Explanation and Elaboration article discusses each checklist item and gives methodological background and published examples of transparent reporting. The STROBE checklist is best used in conjunction with this article (freely available on the Web sites of PLoS Medicine at http://www.plosmedicine.org/, Annals of Internal Medicine at http://www.annals.org/, and Epidemiology at http://www.epidem.com/). Information on the STROBE Initiative is available at www.strobe-statement.org.
